# Marginal Artery of Drummond Masquerading as a Fistulous Tract Resulting in Recurrent Lower Gastrointestinal Bleeding

**DOI:** 10.14309/crj.0000000000001193

**Published:** 2023-11-03

**Authors:** Alexis Bejcek, Anupama Ancha, Tyson Amundsen, Sean Rodich, Steven Smith, Christopher Johnson

**Affiliations:** 1 Division of Gastroenterology, Department of Medicine, Baylor Scott & White Medical Center, Temple, TX; 2 Division of Internal Medicine, Department of Medicine, Baylor Scott & White Medical Center, Temple, TX; 3 Division of Gastroenterology and Hepatology, Department of Medicine, University of Tennessee Health Science Center, Memphis, TN; 4 Department of Radiology, Baylor Scott & White Medical Center, Temple, TX; 5 Division of Gastroenterology, Department of Medicine, Hendrick Medical Center, Abilene, TX

**Keywords:** gastrointestinal bleeding, pseudoaneurysm, pancreatitis, marginal artery of Drummond, coronavirus disease 2019

## Abstract

Lower gastrointestinal bleeding (LGIB) can be caused by a variety of causes. Pseudoaneurysms have been described as a rare etiology of LGIB and are associated with pancreatic pseudocysts that involve adjacent vasculature. Our study describes a 38-year-old man with recent severe coronavirus disease 2019 and necrotizing pancreatitis presenting with hematochezia and blood clots by gastrostomy-jejunostomy. Initial flexible sigmoidoscopy did not elicit an etiology for the LGIB. Recurrent hematochezia prompted colonoscopy and angiography, which demonstrated a pseudoaneurysm in the marginal artery of Drummond as the source. Our case highlights the importance of repeat evaluation of gastrointestinal bleeding of unknown etiology.

## INTRODUCTION

The mortality rate of patients hospitalized with lower gastrointestinal bleeding (LGIB) has been reported at 1.1% in the United States from 2005 to 2014, which has decreased from previous years.^1,2^ Various factors have been shown to increase mortality risk from this condition, including multiple comorbidities, age greater than 70 years, male sex, bleeding from a separate process during hospitalization, coagulation defects, hypovolemia, and anemia requiring blood transfusion. Diverticular bleeding, hemorrhoids, and colorectal polyps have been reported as the most common causes of LGIB.^2^ Several case reports have described gastrointestinal bleeding as a rare manifestation of pseudoaneurysms, often presenting as a complication of pancreatitis.^3–5^ Our study describes a rare case of recurrent LGIB diagnosed as a pseudoaneurysm by endoscopy and angiography.

## CASE REPORT

A 38-year-old man presented to our hospital from a long-term care facility with hematochezia and blood clots by gastrostomy-jejunostomy. He had recently been hospitalized for severe coronavirus disease 2019 with a complicated hospital course in the intensive care unit including necrotizing pancreatitis with an abdominal drain, multiple secondary infections, tracheostomy, and percutaneous endoscopic gastrostomy-jejunostomy. During that hospitalization, he was found to have a pseudoaneurysm of the gastroduodenal artery on computed tomography angiography after a large bloody bowel movement and subsequent drop in hemoglobin. He received embolization of the gastroduodenal and gastroepiploic arteries at that time. He was also diagnosed with hemophagocytic lymphohistiocytosis (HLH), which was confirmed on bone marrow biopsy and responded to dexamethasone.

During transport to our hospital, he was noted to have tachycardia with hypotension requiring norepinephrine and was transfused 1 unit of red blood cells. Hemoglobin was 7.5 g/dL after transfusion. Esophagogastroduodenoscopy was completed and showed a gastrojejunostomy tube in the expected location but was noted to be tight to the mucosa, which was pale in appearance. Flexible sigmoidoscopy revealed localized areas of edematous and erythematous mucosa with some associated oozing throughout the sigmoid colon. A full colonoscopy was not completed because of the presence of solid brown stool beyond 70 cm from the anal verge. There was no elucidated source for upper GI bleeding that would have explained the blood clots initially found in the gastrostomy-jejunostomy. Computed tomography angiography was completed and did not find an etiology of GI bleeding at that time. There was specifically no evidence of diverticulosis on computed tomography imaging before or after this workup. The patient's blood counts began to improve, and he continued dexamethasone therapy for HLH.

**Figure 1.**
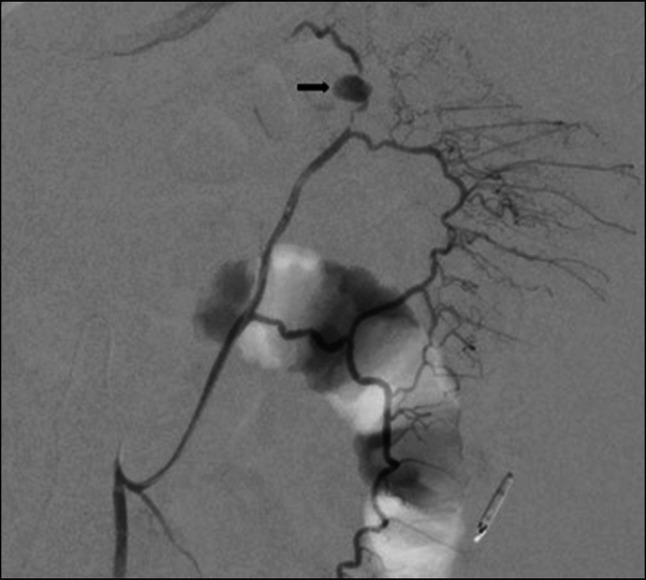
Digital subtraction angiogram with a catheter selected in the ascending branch of the left colic artery demonstrating the pseudoaneurysm (arrow) present immediately distal to the anastomosis with the marginal artery of Drummond.

Repeat evaluation was completed 1 week later because of recurrent hematochezia. Colonoscopy was performed with identification of an apparent fistulous tract with active oozing of blood in the sigmoid colon located at 35 cm. There was a moderate amount of bright red blood in the sigmoid and rectum. Endoscopic clips were placed for identification by interventional radiology with the plan for further evaluation by their team. Angiogram completed by interventional radiology localized a pseudoaneurysm arising from the marginal artery of Drummond just proximal to its anastomosis with the ascending branch of the left colic artery and was successfully embolized (Figure 1). Of note, imaging throughout this time had shown similar severe sequelae of necrotizing pancreatitis, including a multiloculated abscess centered within the pancreatic parenchyma. The component posterior to the spleen showed a possible communication through the left hemidiaphragm to a left lower lobe lung abscess. There were also smaller abscesses within the left posterior pararenal space.

After the embolization, the patient had another acute decrease in hemoglobin caused by a ruptured mesenteric pseudoaneurysm. He had continued to have a clinical decline, including intermittent hypoxia requiring manual bagging and worsening hypotension over several days. After worsening desaturation despite manual bagging, the patient's family ultimately decided to transition to comfort care.

## DISCUSSION

Pseudoaneurysms, such as the one described in this case, have been associated with pancreatitis and can result if a pseudocyst involves adjacent vasculature.^6^ Although rare, it has been seen in up to 10% of patients with chronic pancreatitis.^7^ GI bleeding has been reported as a rare presentation of this condition.^8,9^ This case highlights the importance of repeat endoscopy and angiography in the setting of a GI bleeding of unknown etiology.

Pseudoaneurysms are most commonly asymptomatic but may also present with epigastric pain or GI bleeding.^4,8,9^ GI bleeding, although a common phenomenon, is associated with high morbidity and mortality.^2^ In cases of pseudoaneurysms, bleeding may be intermittent and insidious and can lead to significant blood loss.^8,9^ Given the anatomical location of the pancreas and surrounding splanchnic vessels, bleeding may occur in the bowel, peritoneal cavity, pancreatic duct (hemosuccus pancreaticus), or biliary tree.^10,11^ Hemosuccus pancreaticus is bleeding in the pancreatic duct through the ampulla of Vater into the duodenum and is an extremely rare cause of GI bleeding associated with pseudoaneurysms.^11,12^ In other cases, pseudoaneurysms have been mistaken for malignancy or even carcinomatosis peritonei.^13^

Several case reports have reported mycotic or fungal aneurysm formation in association with HLH.^14,15^ Given that cytokine storm and a marked proinflammatory state are central to the pathophysiology of HLH, further investigation is needed to determine whether HLH predisposes patients, such as ours, to aneurysm/pseudoaneurysm formation.^16^

Pseudoaneurysms prove to be a diagnostic challenge with great heterogenicity of presentation.^3,4,8,9^ Angiography is useful in identifying sources of bleeding and has been used frequently in locating pseudoaneurysms.^3,4,9^ As with other GI bleeds, management should prioritize identification of the source and transfusion if needed.^13^ Embolization serves as the definitive treatment for these cases.^3,17,18^

This case report of pseudoaneurysm of the marginal artery of Drummond after a case of necrotizing pancreatitis presenting with recurrent bleeding highlights the importance of considering pseudoaneurysm formation as a differential of GI bleeding in the correct clinical situation to prevent recurrent procedures and further complications.

## DISCLOSURES

Author contributions: All authors listed above have had substantial contributions to the conception of the work, drafting the work or revising it critically for important intellectual content, final approval of the version to be published, and agreement to be accountable for all aspects of the work.

Financial disclosure: None to report.

Previous presentation: The case was previously presented at the National American College of Gastroenterology Conference October 2022; Charlotte, North Carolina, as a poster presentation.

Informed consent was obtained for this case report.

## References

[1] Charilaou P Devani K Enjamuri D Radadiya D Reddy CM Young M. Epidemiology of lower GI bleed in the United States—an update from the National Inpatient Survey 2005-2014. Am J Gastroenterol 2018;113(Suppl):S319–21.

[2] Strate LL Ayanian JZ Kotler G Syngal S. Risk factors for mortality in lower intestinal bleeding. Clin Gastroenterol Hepatol 2008;6(9):1004–10; quiz 955. 18558513 10.1016/j.cgh.2008.03.021 PMC2643270

[3] Hong GS Wong CY Nambiar R. Massive lower gastrointestinal haemorrhage from a splenic artery pseudoaneurysm. Br J Surg 1992;79(2):174. 1555071 10.1002/bjs.1800790227

[4] Gurala D Polavarapu AD Idiculla PS Daoud M Gumaste V. Pancreatic pseudoaneurysm from a gastroduodenal artery. Case Rep Gastroenterol 2019;13(3):450–5. 31762734 10.1159/000503895 PMC6873056

[5] Carr JA Cho JS Shepard A Nypaver TJ Reddy DJ. Visceral pseudoaneurysms due to pancreatic pseudocysts: Rare but lethal complications of pancreatitis. J Vasc Surg 2000;32(4):722–30. 11013036 10.1067/mva.2000.110055

[6] Bergert H Hinterseher I Kersting S Leonhardt J Bloomenthal A Saeger HD. Management and outcome of hemorrhage due to arterial pseudoaneurysms in pancreatitis. Surgery 2005;137(3):323–8. 15746787 10.1016/j.surg.2004.10.009

[7] Roberts G Friedman AC. Radiology of the liver, biliary tract, pancreas and spleen. Clin Radiol 1988;39(1):201.

[8] Nayyar G Behera A. Gastroduodenal artery pseudoaneurysm as a complication of pancreatitis presenting with lower gastrointestinal bleed. J Clin Diagn Res 2021;15(8):13–4.

[9] Ballinas-Oseguera GA Martínez-Ordaz JL Sinco-Nájera TG Caballero-Luengas C Arellano-Sotelo J Blanco-Benavides R. Management of pseudoaneurysm of the splenic artery: Report of two cases. Cir Cir. 2011;79(3):246–51. 22380996

[10] Stanley JC Wakefield TW Graham LM Whitehouse WM Jr Zelenock GB Lindenauer SM. Clinical importance and management of splanchnic artery aneurysms. J Vasc Surg 1986;3(5):836–40. 3701947

[11] Alshaikhli A Al-Hillan A. Hemosuccus Pancreaticus. StatPearls, Treasure Island (FL), 2022. 34033332

[12] Sakorafas GH Sarr MG Farley DR Que FG Andrews JC Farnell MB. Hemosuccus pancreaticus complicating chronic pancreatitis: An obscure cause of upper gastrointestinal bleeding. Langenbecks Arch Surg 2000;385(2):124–8. 10796050 10.1007/s004230050254

[13] Strate LL Gralnek IM. ACG clinical Guideline: Management of patients with acute lower gastrointestinal bleeding. Am J Gastroenterol 2016;111(5):755–74. 27151132 10.1038/ajg.2016.155 PMC12863135

[14] Varghese B Ting K Lopez-Mattei J Iliescu C Kim J Kim P. Aspergillus endocarditis of the mitral valve with ventricular myocardial invasion, cerebral vasculitis, and intracranial mycotic aneurysm formation in a patient with hemophagocytic lymphohistiocytosis. Med Mycol Case Rep 2018;21:49–51. 29755935 10.1016/j.mmcr.2018.05.001 PMC5944398

[15] Sakamoto K Osumi T Yoshimura S Living-donor liver transplantation providing an adequate chemotherapy for a pediatric patient with anaplastic large cell lymphoma complicated with liver failure due to the aggravation of biliary hepatopathy by secondary hemophagocytic lymphohistiocytosis. Int J Hematol 2020;112(6):900–5. 32710432 10.1007/s12185-020-02949-z

[16] García-Pavón S Yamazaki-Nakashimada MA Báez M Borjas-Aguilar KL Murata C. Kawasaki disease complicated with macrophage activation Syndrome: A Systematic review. J Pediatr Hematol Oncol 2017;39(6):445–51. 28562511 10.1097/MPH.0000000000000872

[17] Bretagne JF Heresbach D Le Jean-Colin I Splenic pseudo- aneurysm rupture into the colon: Colonoscopy before and after successful arterial embolization. Surg Endosc 1987;1(4):229–31. 3455039 10.1007/BF00591154

[18] Baker KS Tisnado J Cho SR Beachley MC. Splanchnic artery aneurysms and pseudoaneurysms: Transcatheter embolization. Radiology 1987;163(1):135–9. 3823426 10.1148/radiology.163.1.3823426

